# The complete plastid genome sequence of *Ilex suaveolens* (H. Lév.) Loes, the most abundant medicinal holly in Mount Huangshan

**DOI:** 10.1080/23802359.2021.1872428

**Published:** 2021-02-09

**Authors:** Lige Yuan, Han Wu, Can Zhang, Ying Wang, Qi Huang, Shiming Fan, Tao Su

**Affiliations:** a Co-Innovation Center for Sustainable Forestry in Southern China, College of Biology and the Environment, Nanjing Forestry University, Nanjing, China; b Key Laboratory of State Forestry Administration on Subtropical Forest Biodiversity Conservation, Nanjing Forestry University, Nanjing, China

**Keywords:** *Ilex suaveolens*, plastid genome, phylogenetic analysis, genetic diversity, conservation

## Abstract

Holly (*Ilex* L.) is a woody dioecious genus cultivated as pharmaceutical, ornamentals, and industrial materials. *Ilex suaveolens* (H. Lév.) Loes is an endemic medicinal holly with a predominant distribution in Mount Huangshan, China. In the present work, the complete plastid genome of *I. suaveolens* was *de novo* sequenced by high-throughput sequencing technology. The newly-assembled plastid genome holds 37.6% of the overall GC content and a length of 157,857 bp, comprising a large single-copy (LSC, 87,255 bp), a small single-copy (SSC, 18,398 bp), and a pair of inverted repeat (IRs, 26,102 bp) regions. The plastid genome annotation suggested the presence of a total of 89 protein-encoding genes, 37 transfer RNA (tRNA) genes, and eight ribosomal RNA (rRNA) genes. The plastome-mediated phylogenetic topology revealed that *I. suaveolens* clustered together with *I. szechwanenesis* and *I. viridis* in the same clade, and a strong relationship between clades and biogeography was found. These data contribute to the understanding of genetic diversity and conservation study of *Ilex* in Mount Huangshan.

Holly (*Ilex* L.), in the monogeneric family of Aquifoliaceae, is a living woody dioecious angiosperm genus, accounting for approximately 700 species (Yao et al. 2016). The majority of *Ilex* genus is used widely due to pharmaceutical, culinary, ornamental, and industrial materials. Approximately 204 *Ilex* species (149 endemic species) have been documented in the China Flora. Given that some systematic studies revealed a high incongruity of phylogenies with the traditional taxonomy, the evolutionary patterns remain to be explored further in *Ilex* (Manen et al. 2002; Yao et al. 2020).

In a transition zone of north-south flora of Eastern China, Mount Huangshan is regarded as a priority spot for biodiversity and conservation. Recent surveys prompted that more than 20 *Ilex* species displayed diversified medicinal properties and economic values (Hao et al. 2013; Qian and Tian 2016; Yi et al. 2016). Among them, *I. suaveolens* is the most proliferous local holly, exhibiting potential functions in scavenging heat, anti-inflammation, and detoxification (Ding et al. 2016). Here, the complete plastid genome of *I. suaveolens* was sequenced, providing essential data for taxonomy and conservation genetics and clues to explore new molecular markers among taxa in Aquifoliaceae (Nock et al. 2011; Zong et al. 2019).

The fresh leaves of *I. suaveolens* were harvested from a 10.24 ha (320 m × 320 m) forest plot (30°8′ 26″ N, 118°6′ 38″ E) in Mount Huangshan (Anhui, China). The dynamic plot ranges in altitude from 430 to 565 m with an annual average temperature of 7.8 °C and annual precipitation of 2394.5 mm. The voucher specimen (YL20190417016) was preserved in the herbarium of Nanjing Forestry University. DNA extraction was conducted according to a previous report (Su et al. 2019), and the next-generation sequencing of the whole-plastid genomes was served by Biodata Biotechnologies Inc. (Hefei, China) on the BGISEQ-500 platform (Shenzhen, China). Approximately 50 MB of high-quality clean paired-end reads was generated, followed by assembling the filtered sequences using SPAdes assembler 3.14.1 software (Bankevich et al. 2012). The plastid genome sequences were further annotated using the DOGMA (Wyman et al. 2004).

The plastid genome of *I. suaveolens* comprised a double-stranded and circular DNA (157,857 bp), containing two inverted repeat (IRs, 26,102 bp) regions separated by a large single-copy (LSC, 87,255 bp) and a small single-copy (SSC, 18,398 bp) sections. The overall GC content is 37.6%, and the corresponding values in LSC, SSC, and IRs regions are 35.7%, 31.9%, and 42.9%, respectively. The plastid genome was predicted to encode 134 genes, including 89 protein-coding genes, 37 tRNA genes, and eight rRNA genes. Seven protein-coding genes, eight tRNA genes, and four rRNA genes show duplications in IR regions. Nineteen genes were identified to have two exons, and two genes (*clpP* and *ycf3*) contained three exons. Using MAFFT v7.471, the multiple sequences of 15 *Ilex* species were aligned (Katoh et al. 2019). The plastid topology of phylogenies was reconstructed using the software MEGA X, showing that *I. suaveolens* is mostly related to *I. szechwanensis* and *I. viridis* in the clade III (Figure 1). In summary, the plastid phylogenetic tree displayed superior resolution for species discrimination and a better indication of the phylogeographic distribution in *Ilex*.

**Figure 1.**
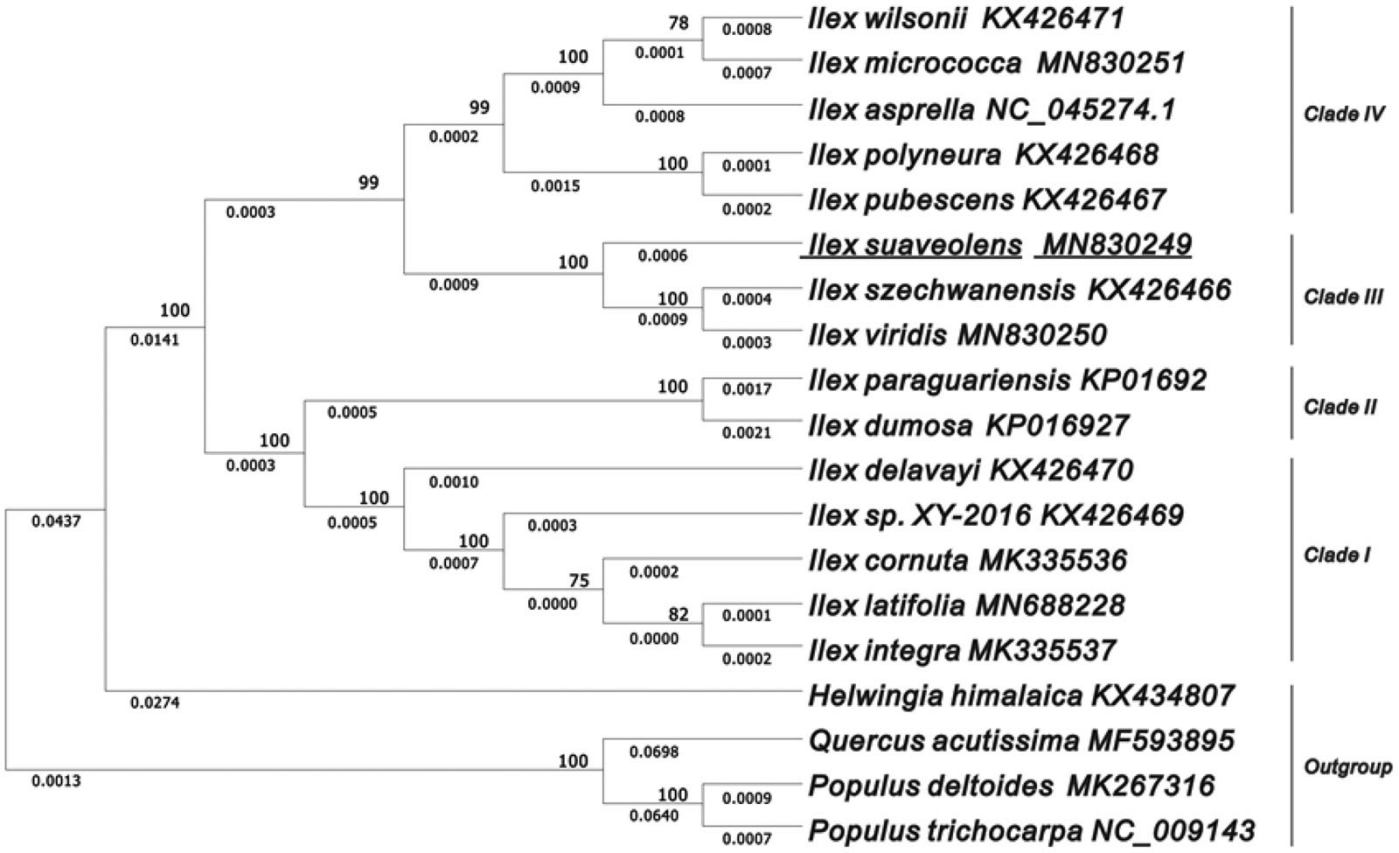
The evolutionary tree was constructed by MEGA X using the Maximum Likelihood method and the Tamura-Neighbour model. The percentage of phylogenetic trees associated with taxa is shown next to the branches. The analyses involved 15 plastomes of *Ilex* species with *P. trichocarpa*, *P. deltoides*, *Q. acutissima*, and *H. himalaica* rooted as the outgroup. The bootstrap values are shown on the branches of the phylogenetic tree based on 1000 replicates.

## Data Availability

The complete plastid genome data that support the findings of this study are openly available in the GenBank of NCBI (https://www.ncbi.nlm.nih.gov/) under the accession number of MN830249. The raw sequence reads have been deposited in GSA database (https://bigd.big.ac.cn/gsa/) associated with the accession number of CRR147931.
